# Regulation of the Abundance of Kaposi’s Sarcoma-Associated Herpesvirus ORF50 Protein by Oncoprotein MDM2

**DOI:** 10.1371/journal.ppat.1005918

**Published:** 2016-10-03

**Authors:** Tzu-Hsuan Chang, Shie-Shan Wang, Lee-Wen Chen, Ying-Ju Shih, Li-Kwan Chang, Shih-Tung Liu, Pey-Jium Chang

**Affiliations:** 1 Graduate Institute of Biomedical Sciences, College of Medicine, Chang-Gung University, Taoyuan, Taiwan; 2 Department of Pediatric Surgery, Chang-Gung Memorial Hospital, Chiayi, Taiwan; 3 Department of Respiratory Care, Chang-Gung University of Science and Technology, Chiayi, Taiwan; 4 Graduate Institute of Clinical Medical Sciences, College of Medicine, Chang-Gung University, Taoyuan, Taiwan; 5 Department of Biochemical Science and Technology, College of Life Science, National Taiwan University, Taipei, Taiwan; 6 Department of Microbiology and Immunology, College of Medicine, Chang-Gung University, Taoyuan, Taiwan; 7 Department of Nephrology, Chang-Gung Memorial Hospital, Chiayi, Taiwan; Keck School of Medicine of the University of Southern California, UNITED STATES

## Abstract

The switch between latency and the lytic cycle of Kaposi’s sarcoma-associated herpesvirus (KSHV) is controlled by the expression of virally encoded ORF50 protein. Thus far, the regulatory mechanism underlying the protein stability of ORF50 is unknown. Our earlier studies have demonstrated that a protein abundance regulatory signal (PARS) at the ORF50 C-terminal region modulates its protein abundance. The PARS region consists of PARS-I (aa 490–535) and PARS-II (aa 590–650), and mutations in either component result in abundant expression of ORF50. Here, we show that ORF50 protein is polyubiquitinated and its abundance is controlled through the proteasomal degradation pathway. The PARS-I motif mainly functions as a nuclear localization signal in the control of ORF50 abundance, whereas the PARS-II motif is required for the binding of ubiquitin enzymes in the nucleus. We find that human oncoprotein MDM2, an ubiquitin E3 ligase, is capable of interacting with ORF50 and promoting ORF50 degradation in cells. The interaction domains between both proteins are mapped to the PARS region of ORF50 and the N-terminal 220-aa region of MDM2. Additionally, we identify lysine residues at positions 152 and 154 in the N-terminal domain of ORF50 critically involved in MDM2-mediated downregulation of ORF50 levels. Within KSHV-infected cells, the levels of MDM2 were greatly reduced during viral lytic cycle and genetic knockdown of MDM2 in these cells favored the enhancement of ORF50 expression, supporting that MDM2 is a negative regulator of ORF50 expression. Collectively, the study elucidates the regulatory mechanism of ORF50 stability and implicates that MDM2 may have a significant role in the maintenance of viral latency by lowering basal level of ORF50.

## Author Summary

The switch of Kaposi’s sarcoma-associated herpesvirus (KSHV) from latency to lytic replication is an essential process for controlling viral dissemination and the pathogenesis of KSHV-associated diseases. The virally encoded ORF50 protein is known as the master controller of viral reactivation. We previously reported that a two-component PARS region, which contains PARS-I and PARS-II, in ORF50 influences its protein abundance. In this study, we elucidate the regulatory mechanism of the ORF50 protein stability. We find that degradation of ORF50 involves at least two critical steps, including nuclear translocation and the subsequent degradation in the nucleus. The PARS-I motif is responsible for the nuclear translocation of ORF50, whereas the PARS-II motif is required for ORF50 degradation in the nucleus. Furthermore, we show that the PARS region is the primary docking site for cellular oncoprotein MDM2, an E3 ubiquitin ligase. Direct interaction of MDM2 with ORF50 promotes ORF50 degradation. The putative ubiquitin acceptor sites targeted by MDM2 are mapped to K152 and K154 in the N-terminal region of ORF50. In the context of KSHV-infected cells, we also provide evidence that MDM2 is involved in the regulation of the ORF50 expression.

## Introduction

Kaposi’s sarcoma-associated herpesvirus (KSHV), also referred to as human herpesvirus-8 (HHV-8), is the etiologic agent of Kaposi’s sarcoma (KS), primary effusion lymphomas (PELs) and multicentric Castleman’s disease [1–3]. Numerous lines of evidence have shown that both KSHV latent and lytic phases of its life cycle are required for the pathogenesis of viral associated diseases [4]. The switch of the virus from latency to the lytic cycle is controlled by the expression of a transcription activator encoded by open reading frame 50 (ORF50) of the viral genome [5, 6]; ectopic expression of which is sufficient to disrupt viral latency and drive the lytic cascade to completion [7].

**Fig 1:**
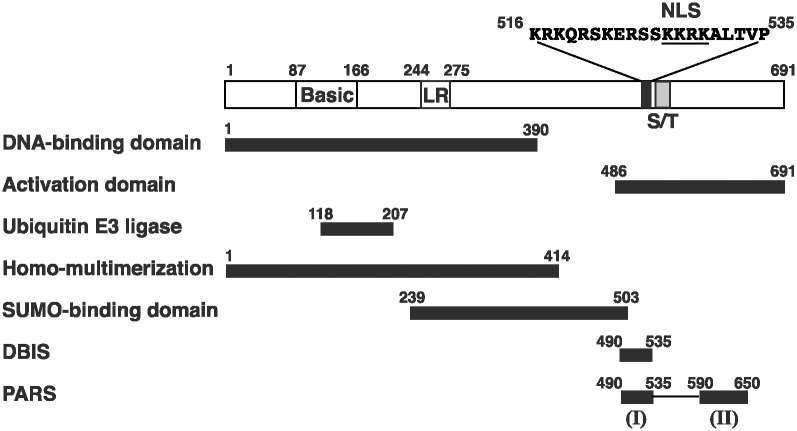
Structural and functional domains of ORF50 protein. Numbers in the diagram correspond to amino acid positions. NLS, nuclear localization signal; Basic, basic-rich region; LR, leucine heptapeptide repeat; S/T, serine/threonine-rich region. Distinct functional domains or regulatory motifs are shown, including the DNA-binding domain, activation domain, ubiquitin E3 ligase domain, homo-multimerization domain, SUMO-binding domain, DNA-binding inhibitory sequence (DBIS), and protein abundance regulatory signal (PARS). The PARS contains two components located in aa 490–535 (PARS-I) and aa 590–650 (PARS-II), respectively.

ORF50 protein, also called RTA (replication and transcription activator), is a multifunctional protein, which transcriptionally activates many KSHV or cellular genes and is involved in the assembly of replication complexes required for lytic DNA synthesis [8]. Besides functioning as a transcription and replication activator, ORF50 also has an ubiquitin E3 ligase activity [9]. ORF50 is 691 amino acids (aa) long in which multiple functional domains and regulatory motifs have been identified (Fig 1). Like a typical transcription activator, ORF50 has both a DNA-binding domain (aa 1–390) and an activation domain (aa 486–691), which are located in the N-terminal and C-terminal regions, respectively [10, 11]. Earlier studies showed that nuclear translocation of ORF50 protein is mediated through a nuclear localization signal, KKRK, located between aa 527 and 530 [12]. Although another lysine-rich sequence located between aa 6 and 12 has also been implicated in nuclear localization, the function of this sequence as an NLS in the context of full-length ORF50 cannot be verified [12–14]. Within the cell, ORF50 is hyperphosphorylated and may form multimers [15]. The ORF50’s homo-multimerization domain has been mapped to the N-terminal 414-aa region that encompasses a leucine heptapeptide repeat between aa 244 and 275 [15]. Studies from Yu et al. [9] showed that a RING-like ubiquitin E3 ligase domain is located near the N-terminal region from aa 118 to 207 (Fig 1). Izymiya et al. [16] also reported that ORF50 has multiple small ubiquitin-like modifier (SUMO)-interacting motifs (SIMs) distributed in the region between aa 239 and 503 (Fig 1). Due to the presence of the intrinsic ubiquitin E3 ligase activity and the capacity to bind SUMOs with high affinity, ORF50 is suggested to be a SUMO-targeting ubiquitin ligase (STUBL) [16]. We have previously identified two regulatory motifs within the C-terminal region, which independently influence different functions of ORF50 [10, 17]. One motif is called DNA-binding inhibitory sequence (DBIS), which represses the DNA-binding activity of ORF50. The other regulatory motif is named PARS (protein abundance regulatory signal) that controls the protein abundance of ORF50. The PARS is composed of two sub-components: PARS-I and PARS-II. PARS-I region partially overlaps DBIS (aa 490–535), whereas the PARS-II region is located between aa 590 and 650 (Fig 1).

ORF50 is unstable since mutating specific regions in the protein markedly increases its abundance [10, 17]. Although Yu et al. [9] and Izymiya et al. [16] showed that ORF50 possesses ubiquitin E3 ligase activity and targets itself or several cellular proteins for polyubiquitination *in vitro*, it is unclear whether ORF50 autoregulates its own stability via its E3 ligase function in cells. In fact, several ORF50 mutants defective in the E3 ligase activity are not present in higher amounts than the wild-type ORF50 [9], suggesting that the E3 ligase activity may be unrelated to its own stability. Our previous studies showed that mutating either PARS-I or PARS-II leads to abundant expression of ORF50. Additionally, when the PARS region is fused to glutathione S-transferase, the abundance of the fusion protein decreases as well [10, 17]. Despite the importance of the PARS region in controlling the ORF50 abundance, so far, how PARS affects protein abundance is unknown.

MDM2 is an oncoprotein with ubiquitin E3 ligase activity, which negatively modulates the functions of p53 tumor suppressor by promoting p53’s ubiquitination and degradation by 26S proteasome [18]. Previously, Sarek et al. [19] and Ye et al. [20] showed that Nutlin-3, an MDM2 inhibitor, could effectively inhibit the growth of KSHV-associated tumors in their xenograft mouse models. Particularly, Ye et al. [20] noticed that substantial activation of viral lytic genes was concomitantly observed after treatment with Nutlin-3 in the KS xenografts. Recent studies by Balistreri et al. [21] also revealed that MDM2 siRNA activates KSHV reactivation. These findings suggest that MDM2 is a negative regulator of KSHV lytic gene expression.

In this study, we characterized the function of each PARS component in controlling the ORF50 abundance and determined the molecular mechanism underlying the protein stability of ORF50. We found that the PARS-I motif serves as an NLS in the control of ORF50 stability, and the PARS-II motif is required for ORF50 degradation after the protein enters the nucleus. Importantly, we showed that MDM2 binds to the PARS region of ORF50 and promotes its degradation. The biological significance of MDM2 in establishing or maintaining KSHV latency in infected cells is discussed.

## Results

### Nuclear translocation and the abundance of ORF50

**Fig 2:**
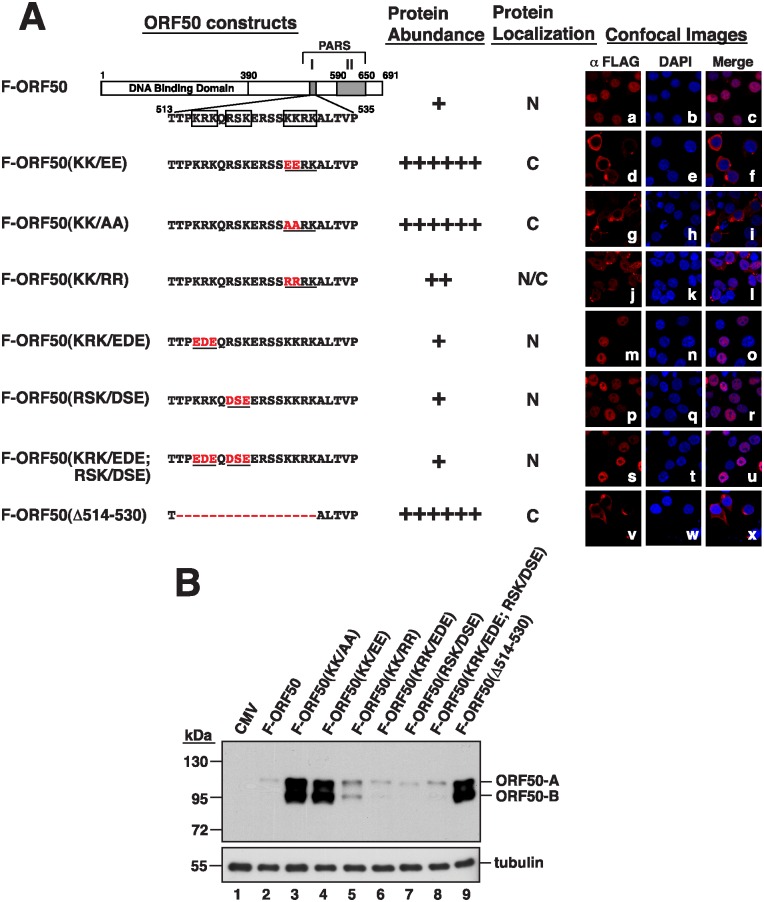
Correlation between the subcellular localization and protein abundance of PARS-I mutants. (A) Left, schematic diagram of PARS-I mutants of ORF50. Specific deletion or amino acid substitutions in the PARS-I motif are indicated in red color in the diagram. The protein abundance and subcellular localization of each PARS-I mutant protein are summarized. The degree of protein abundance is indicated by “+”. N: nucleus; C: cytoplasm; N/C: both nucleus and cytoplasm. Right, confocal microscopy images of PARS-I mutant proteins in 293T cells. (B) Abundance of PARS-I mutants in 293T cells. Cells were transfected with the indicated plasmids encoding FLAG-tagged ORF50 mutants for 24 hr. Cell lysates were then immunoblotted using anti-FLAG antibody. ORF50-A: hyperphosphorylated ORF50; ORF50-B: hypophosphorylated ORF50.

We previously found that deleting PARS-I or PARS-II (Fig 1) increases the abundance of ORF50 [10, 17]. Therefore, in this study, we first investigated the mechanism by which PARS-I influences the abundance of ORF50. There are three basic amino acid motifs in PARS-I, including KRK (aa 516–518), RSK (aa 520–522) and KKRK (aa 527–530) (Fig 2A). We found that after mutating the KRK residues in Flag-tagged ORF50 (F-ORF50), from aa 516 to 518, to EDE (KRK/EDE) (Fig 2A), the protein was found in the nucleus under a microscope (Fig 2Am–2Ao). Immunoblotting showed that the protein was expressed at a level similar to that of the wild-type F-ORF50 (Fig 2B, lanes 2 and 6). Mutating the RSK residues, from aa 520 to 522 (Fig 2A), to DSE (RSK/DSE) or mutating both KRK and RSK (KRK/EDE; RSK/DSE) (Fig 2A) also did not change the localization and abundance of F-ORF50 in the cell (Fig 2Ap–2Au and 2B, lanes 7 and 8). However, after changing the KKRK sequence to EEKK (KK/EE) or to AARK (KK/AA) (Fig 2A), the mutant proteins were no longer present in the nucleus (Fig 2Ad–2Ai), verifying that the KKRK sequence of ORF50 is the NLS [14]. Meanwhile, immunoblot analysis revealed that the amounts of F-ORF50(KK/EE) and F-ORF50(KK/AA) in the lysates were substantially higher than that of F-ORF50 (Fig 2, lanes 2–4). In these two mutants, two ORF50 forms, ORF50-A and ORF50-B, were abundantly produced in cells. OR50-A was verified as a hyperphosphorylated form, and ORF50-B was a hypophosphorylated form (S1 Fig). We also mutated the NLS from KKRK to RRRK (KK/RR) (Fig 2A), and found that the mutant protein was present in both the cytoplasm and the nucleus (Fig 2Aj–2Al). Immunoblotting showed that the amount of F-ORF50(KK/RR) expressed by the cells was higher than that of F-ORF50 but less than that of F-ORF50(Δ514–530), a mutant F-ORF50 having its PARS-I deleted (Fig 2B, lane 5). Noteworthily, due to dramatic differences in the protein abundance between wild-type ORF50 and its mutants in cells, the confocal microscopic images shown in Fig 2A were taken using different microscopic settings by which the intensities of fluorescence signals were similar between wild-type ORF50 and its mutants. The images that were captured using the same settings on the confocal microscopy are shown in S2 Fig. In these images (S2 Fig), the fluorescence intensities of ORF50 and its mutants in cells visualized by confocal microscopy are highly correlated with their levels of protein expression detected by immunoblotting (Fig 2B).

**Fig 3:**
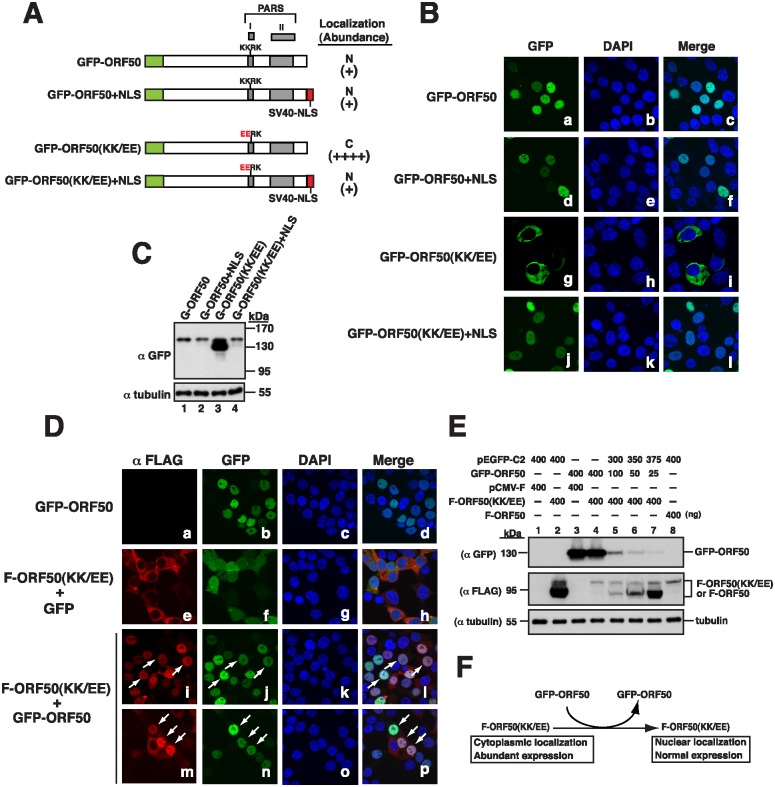
Nuclear translocation of ORF50(KK/EE) and its abundance. (A) Schematic diagram of GFP-ORF50 and GFP-ORF50(KK/EE) constructs with or without an appendage of the SV40 NLS. The subcellular localization and protein abundance of each GFP fusion construct determined by confocal microscopy (B) and by immunoblot analysis (C) are summarized. (D) Confocal images of both F-ORF50(KK/EE) and GFP-tagged proteins expressed in 293T cells. Cells were cotransfected with plasmids expressing F-ORF50(KK/EE) and GFP or GFP-ORF50 for 24 hr. Intracellular localization of F-ORF50(KK/EE) (red) and GFP or GFP-ORF50 (green) in cells was analyzed by confocal microscopy. Arrows indicate the cells expressing both F-ORF50(KK/EE) and GFP-ORF50. (E) Changes in the expression of F-ORF50(KK/EE) by coexpression with GFP-ORF50. Various amounts of the GFP-ORF50 expression plasmid (0, 25, 50, 100 and 400 ng) were cotransfected with 400 ng of the F-ORF50(KK/EE) expression plasmid into 293T cells. The expression levels of F-ORF50(KK/EE) and GFP-ORF50 in cells were determined by immunoblotting using anti-FLAG and anti-GFP antibody, respectively. The expression of wild-type F-ORF50 in 293T cells was also included in the experiment (lane 8). (F) Summary of phenotypic changes of F-ORF50(KK/EE) in the presence of GFP-ORF50.

We also linked the NLS from the simian virus 40 (SV40) large T antigen to the C-terminus of GFP-ORF50 and GFP-ORF50(KK/EE), two green fluorescent protein (GFP) tagging constructs (Fig 3A). As expected, GFP-ORF50(KK/EE) was present in the cytoplasm (Fig 3Bg–3Bi) and expressed abundantly in cells (Fig 3C, lane 3). However, we found that the protein linked to the NLS, GFP-ORF50(KK/EE)+NLS, entered the nucleus (Fig 3Bj–3Bl) and was expressed at a level comparable to that of GFP-ORF50 or GFP-ORF50+NLS (Fig 3C, lanes 1, 2 and 4). Taken together, these results showed that nuclear entry decreases the abundance of ORF50.

### Nuclear translocation of F-ORF50(KK/EE) and its abundance

Since OFR50 oligomerizes [15], this study further investigated whether the oligomerization of ORF50(KK/EE) with wild-type ORF50 affects the localization and abundance of ORF50(KK/EE). Therefore, we cotransfected 293T cells with plasmids that express F-ORF50(KK/EE) and GFP-ORF50. Confocal laser-scanning microscopy showed that both GFP-ORF50 and F-ORF50(KK/EE) were present in the nucleus (Fig 3Di–3Dp). A parallel experiment showed that F-ORF50(KK/EE) was present in the cytoplasm when the cells were cotransfected with plasmids that express GFP and F-ORF50(KK/EE) (Fig 3De–3Dh), verifying that F-ORF50(KK/EE) is transported into the nucleus through its interaction with GFP-ORF50. We also found that the amounts of F-ORF50(KK/EE) expressed from 400 ng plasmid decreased when 25–400 ng pEGFP-ORF50 was cotransfected in a dose-dependent manner (Fig 3E, lanes 4–7). Our results therefore revealed that two mutant phenotypes of F-ORF50(KK/EE), including subcellular localization and protein abundance, could be rescued by coexpression with GFP-ORF50 (Fig 3F).

### Influence of PARS-II on the abundance of ORF50

**Fig 4:**
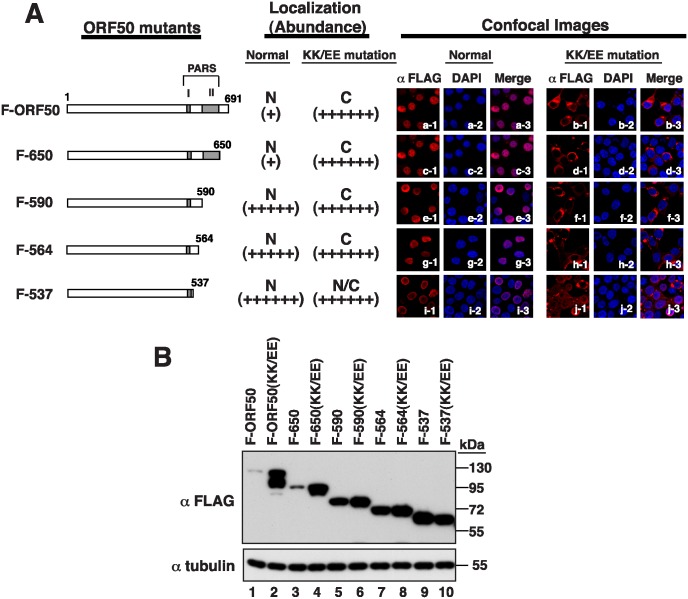
The PARS-II motif functions in the nucleus in the control of ORF50 abundance. (A) Left, diagram of PARS-I and/or PARS-II mutants of ORF50. A series of ORF50 C-terminal deletions with or without the KK-to-EE mutation in the PARS-I motif were included. All ORF50 mutants contain a FLAG tag at their N terminus. The subcellular localization and protein abundance of each of ORF50 mutants in 293T cells are summarized in the diagram. The degree of protein abundance is indicated by “+”. N: nucleus; C: cytoplasm; N/C: both nucleus and cytoplasm. Right, representative confocal images showing the subcellular localization of these ORF50 mutants. (B) Immunoblot analysis of ORF50 deletion mutants in 293T cells.

We created C-terminal deletions in F-ORF50 and F-ORF50(KK/EE), from amino acid 650, 590, 564, and 537 (F-650, F-590, F-564, and F-537, respectively), to study the action of PARS-II (Fig 4A). Confocal microscopy revealed that, as expected, the F-ORF50(KK/EE) mutants with these deletions were present in the cytoplasm (Fig 4Ad, 4Af, 4Ah and 4Aj). Immunoblot analysis also revealed that these proteins were expressed abundantly at levels comparable to that of F-ORF50(KK/EE) (Fig 4B, lanes 2, 4, 6, 8 and 10), which is consistent with the results that ORF50’s abundance is high when it is retained in the cytoplasm. On the other hand, deleting the PARS-II region from F-ORF50, i.e. F-590, F-564 and F-537, substantially enhanced the protein abundance (Fig 4B, lanes 5, 7 and 9). Remarkably, these PARS-II deletion mutants were exclusively localized in the nucleus (Fig 4Ae, 4Ag and 4Ai). Although the PARS-II region was not required for the nuclear translocation of ORF50, it is involved in the subsequent control of ORF50 abundance in the nucleus. Notably, the confocal images shown in Fig 4A were taken using different microscope settings to show the localization of ORF50 and its mutants. The images that were captured using the same microscopic settings are shown in S3 Fig.

### Phenotypic changes of F-ORF50(KK/EE) induced by PARS-II mutants

**Fig 5:**
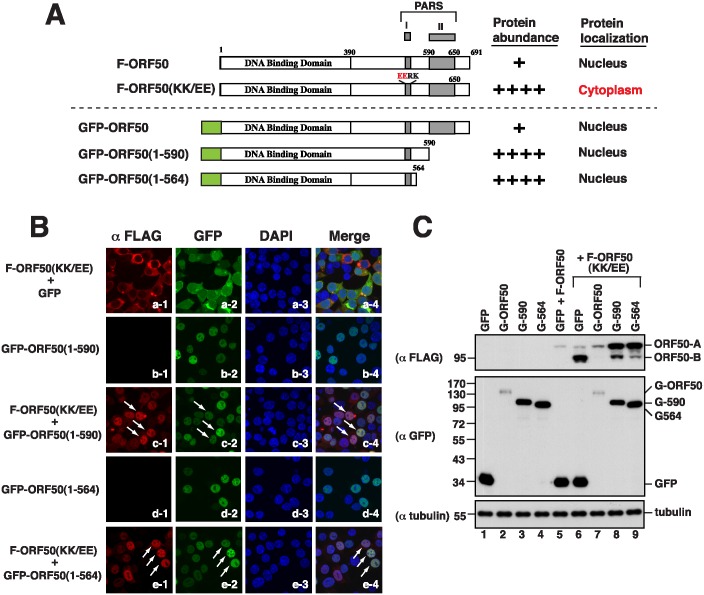
Phenotypic changes of F-ORF50(KK/EE) by different GFP-ORF50 deletion effectors. (A) Diagram of GFP-ORF50 deletion constructs and a summary of their characteristics. (B) Confocal images of 293T cells coexpressing F-ORF50(KK/EE) and GFP-ORF50 mutants. The subcellular localization of both F-ORF50(KK/EE) (red) and GFP-tagged proteins (green) in transfected cells was analyzed by confocal microscopy. (C) Effect of GFP-ORF50 deletion mutants on the expression of F-ORF50(KK/EE). 293T cells were cotranfected with equal amounts (400 ng) of plasmids encoding F-ORF50(KK/EE) and the indicated GFP-tagged proteins. Cell lysates were immunoblotted with either anti-FLAG antibody or anti-GFP antibody.

Besides wild-type GFP-ORF50, we tested whether different PARS-II deletion effectors, including GFP-ORF50(1–590) and GFP-ORF50(1–564), could rescue the subcellular localization and protein abundance of F-ORF50(KK/EE) (Fig 5). Both GFP-ORF50(1–590) and GFP-ORF50(1–564) effectors alone were localized in the nucleus of cells (Fig 5A and 5B) and expressed at levels higher than that of GFP-ORF50 (Fig 5A and 5C). We found that cotransfecting 293T cells with plasmids that express F-ORF50(KK/EE) and GFP-ORF50(1–590) also caused nuclear translocation of F-ORF50(KK/EE) (Fig 5Bc1–Bc4). However, the nuclear translocation of F-ORF50(KK/EE) conveyed by GFP-ORF50(1–590) could not completely reduce the abundance of F-ORF50(KK/EE) to its wild-type level (Fig 5C, lanes 5–8). Immunoblotting revealed that although the amount of hypophosphorylated F-ORF50(KK/EE) (B form) was markedly reduced in the presence of GFP-ORF50(1–590), we found that the hyperphosphorylated F-ORF50(KK/EE) (A form) was inversely increased under the condition (Fig 5C, lanes 6 and 8). A similar result was also observed when cells were cotransfected with plasmids that encode GFP-ORF50(1–564) and F-ORF50(KK/EE) (Fig 5Be1–5Be4 and 5C, lane 9). These results emphasize that nuclear entry is not the only factor affecting the abundance of ORF50; PARS-II is also required to modulate ORF50 abundance in the nucleus.

### Ubiquitination of ORF50

**Fig 6:**
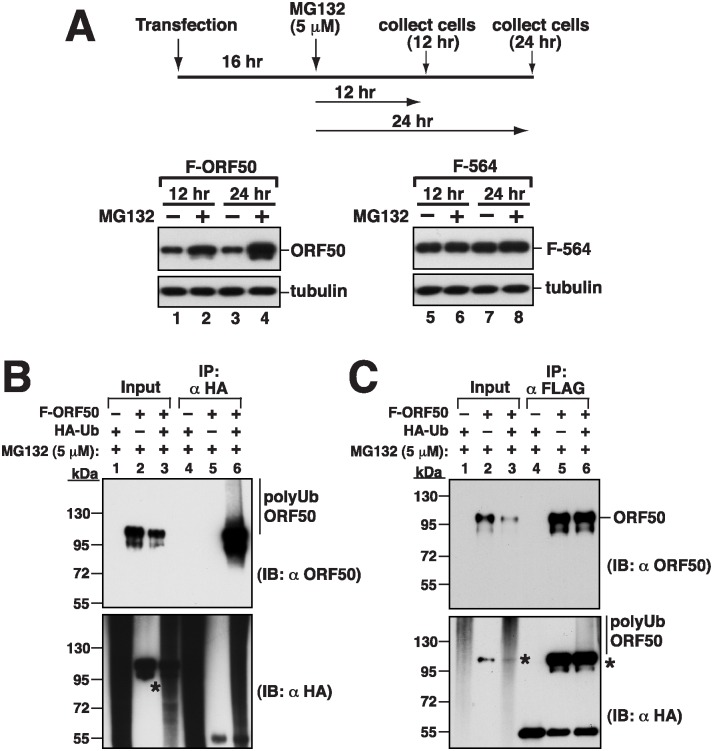
The protein abundance of ORF50 is controlled through the ubiquitin-proteasomal degradation pathway. (A) Effect of MG132 on ORF50 abundance in cells. 293T cells were transfected with a plasmid encoding F-ORF50 or F-ORF50(1–564). At 16 hr after transfection, cells were untreated or treated with 5 μM of MG132 for another 12 hr and 24 hr. Cell lysates were analyzed by immunoblotting using anti-FLAG antibody. (B and C) Ubiquitination of ORF50 in cells. 293T cells were transfected with plasmids encoding HA-ubiquitin (HA-Ub) or/and F-ORF50. At 16 hr posttransfection, cells were treated with MG132 for another 24 hr. Denatured lysates were immunoprecipitated (IP) with either anti-HA antibody (B) or anti-FLAG antibody (C). Cell lysates (input) and the immunoprecipitated proteins were analyzed by immunoblotting (IB) using anti-ORF50 or anti-HA antibody. Asterisks indicate the cross-reaction of ORF50 with the used antibodies (S4 Fig).

We further investigated whether the abundance of ORF50 is attributed to the control by the ubiquitin-proteasome pathway. We transfected 293T cells with pCMV-FLAG-ORF50 to express F-ORF50, and then treated the cells with a 26S proteasome inhibitor, MG132, at 16 h after transfection. In an immunoblot study, we found that the amount of F-ORF50 in the cells that were treated with MG132 was higher than the protein from the cells untreated with MG132 (Fig 6A, lanes 1 and 2); the difference increased further at 24 h after MG132 treatment (Fig 6A, lanes 3 and 4). We also transfected 293T cells with pCMV-FLAG-ORF50(1–564), which expresses a protein that contains the N-terminal 564 amino acids in ORF50, F-564. We found that the protein was present in the cells abundantly and MG132 treatment did not increase its abundance (Fig 6A, lanes 5–8), showing that the region from aa 565 to the C-terminus, including PARS-II, influences 26S proteasome-mediated degradation of ORF50. We also cotransfected 293T cells with plasmids that express hemagglutinin-tagged ubiquitin (HA-Ub) and F-ORF50 and treated the cells with MG132. Cells were then lysed under denaturing conditions and proteins in the lysate were immunoprecipitated with anti-HA or anti-FLAG antibody (Fig 6B and 6C). Immunoblot analysis using anti-ORF50 antibody revealed polyubiquitinated ORF50 (Fig 6B, lanes 6). Polyubiquitinated F-ORF50 was also detected in the lysate by immunoblotting with anti-HA antibody (Fig 6C, lane 6), showing that ORF50 is polyubiquitinated in cells. During the course of experiments, we found that several commercially available anti-immunoglobulin secondary antibodies cross-reacted with overexpressed ORF50 in immunoblotting experiments (asterisks in Fig 6B and 6C). The epitope of ORF50 protein recognized by these secondary antibodies has been mapped to the C-terminal region from aa 650 to 691 (S4 Fig). Despite the significant rate of the cross-reaction between secondary antibodies and ORF50, it does not affect our main conclusions in the study.

### Delineating the regions in ORF50 that are required for ubiquitination

**Fig 7:**
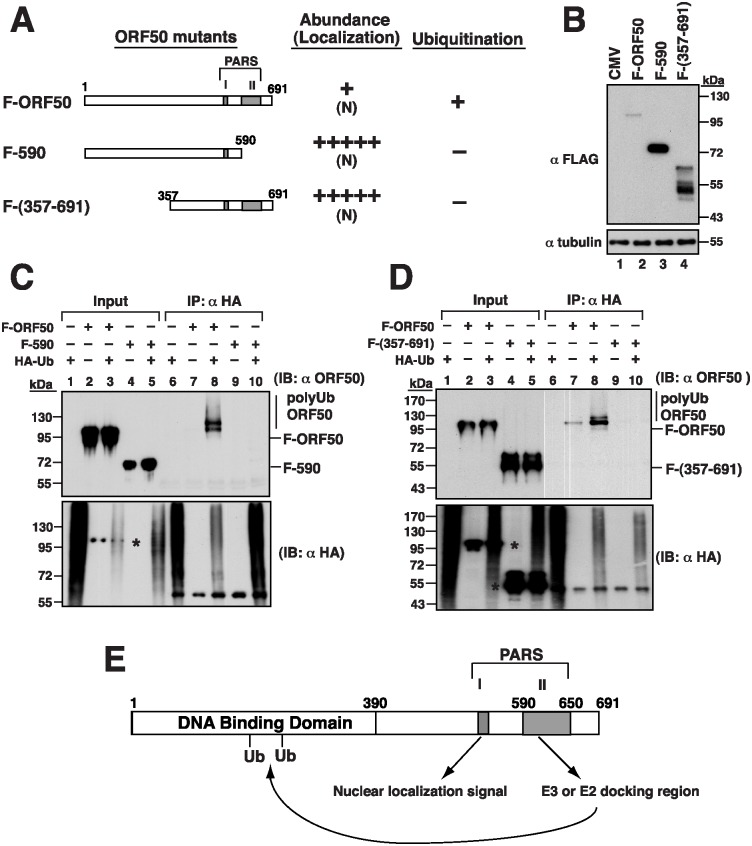
Both N-terminal and C-terminal regions in ORF50 are required for ubiquitination. (A) Deletion constructs of ORF50 and a summary of their intracellular localization, protein abundance and ubiquitination status. (B) Immunoblot analysis of F-ORF50, F-ORF50(1–590) and F-ORF50(357–691) in 293T cells. (C and D) Mutants F-ORF50(1–590) and F-ORF50(357–691) were evaluated for ubiquitination in 293T cells. Cells were transfected with the indicated plasmids. At 16 hr after transfection, cells were treated with MG132 for another 24 hr. Cell lysates were immunoprecipitated and immunoblotted as described in Fig 6B. Asterisks indicate the cross-reaction of ORF50 with the used antibodies (S4 Fig). (E) Proposed model for ORF50 ubiquitination. The PARS-I is responsible for the nuclear translocation of ORF50, and the PARS-II motif is required for the binding of specific ubiquitin enzymes. The ubiquitin acceptor sites are likely to be located in the N-terminal 356-aa region.

To further map the critical regions in ORF50 that are responsible for ubiquitination, we first tested the PARS-II deletion mutant F-590 (Fig 7A). 293T cells were cotransfected with plasmids that express HA-Ub and F-ORF50 or F-590. The cell lysates were then immunoprecipitated using anti-HA antibody, followed by immunoblot analysis with anti-ORF50 antibody (Fig 7C). Smear ubiquitinated ORF50 bands were detected only if the cells were cotransfected with plasmids that express F-ORF50 and HA-Ub (Fig 7C, lane 8), but not if the cells were cotransfected with plasmids that express F-590 and HA-Ub (Fig 7C, lane 10) or if the cells were not cotransfected with the HA-Ub-expressing plasmid (Fig 7C, lanes 7 and 9). These results showed that ORF50 is not ubiquitinated after the region containing PARS-II is deleted. Due to the fact that the C-terminal region from aa 590 to 691 does not contain lysine residues, we analyzed the possible involvement of the N-terminal portion of ORF50 in ubiquitination. Sequence analysis showed that 19 out of 25 lysine residues are distributed within the N-terminal 356-aa region of ORF50. After deleting the N-terminal 356-aa region, we found that the resultant F-ORF50(357–691) was localized in the nucleus and the amount of the protein in cells was much higher than that of F-ORF50 (Fig 7A and 7B, lanes 2 and 4). A similar cotransfection study was conducted using the plasmids expressing HA-Ub and F-ORF50(357–691). Although F-ORF50(357–691) was present in the cell lysates after transfection (Fig 7D, lanes 4 and 5), it was not immunoprecipitated by anti-HA antibody and detected by anti-ORF50 antibody (Fig 7D, lanes 9 and 10), indicating that ORF50 is not ubiquitinated without its N-terminal region. These results suggested that both the N-terminal and C-terminal regions are required for ORF50 ubiquitination (Fig 7E and see discussion below), even though the C-terminal region from aa 590 to 691 does not contain a lysine residue.

### MDM2 and the degradation of ORF50

**Fig 8:**
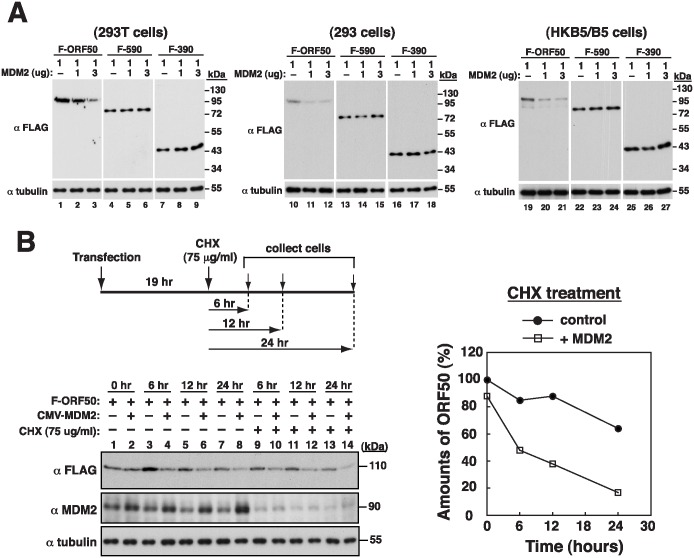
Human MDM2 promotes ORF50 degradation. (A) Effect of MDM2 expression on ORF50 abundance. Increasing amounts of an MDM2 expression plasmid, pCMV6-XL-MDM2, were cotransfected with a plasmid expressing F-ORF50, F-590 or F-390 in 293T, 293 or HKB5/B5 cells. At 24 hr after cotransfection, the expression of these ORF50 proteins was examined by immunoblotting. (B) Evaluation of the half-life of ORF50 in MDM2-transfected cells. 293T cells were cotransfected with an F-ORF50 expression plasmid and pCMV6-XL-MDM2 or control vector. At 19 hr after transfection, cells were untreated or treated with cycloheximide for another 6, 12 and 24 hr. The expression of F-ORF50 and MDM2 were analyzed by immunoblotting with anti-FLAG and anti-MDM2 antibody, respectively. The relative levels of F-ORF50 from immunoblots (left panel) were quantified by densitometry and normalized to tubulin, which are depicted in the right panel.

To identify the E3 ubiquitin ligase of ORF50, the possible roles of cullin-RING E3 ligases, the largest family of ubiquitin ligases [22], in the control of ORF50 protein abundance have been investigated. However, we did not find an elevated F-ORF50 expression after coexpression with individual dominant-negative cullins in 293T cells (S5A and S5B Fig). Besides cullin-based E3 ligases, we have tested several ubiquitin E3 ligases, including MDM2, RNF4, or UBE3A (also known as E6-AP), for their ability to promote ORF50’s degradation in a transfection study. After cotransfecting 293T cells to express both F-ORF50 and these E3 ligases, only the expression of MDM2 reduced the steady-state levels of F-ORF50 (Fig 8A, lanes 1–3; S5C and S5D Fig). However, under the same conditions, overexpression of MDM2 did not influence the levels of F-590 and F-390 (Fig 8A, lanes 4–9). Similar results were also observed in 293 cells and in HKB5/B5 cells (Fig 8A, lanes 10–27). We also found that knockdown of MDM2 in 293T cells increased levels of F-ORF50, but not F-590, in a transient transfection experiment (S6 Fig). To demonstrate whether the reduced expression of F-ORF50 by MDM2 was attributed to protein degradation, we examined the half-life of F-ORF50 by treating cells with cycloheximide, a protein synthesis inhibitor, in 293T cells. We found that both the steady-state level and half-life of F-ORF50 were significantly reduced in cells over-expressing MDM2 as compared with control cells (Fig 8B). These results strongly suggested that MDM2 triggers the degradation of ORF50.

### Interaction between MDM2 and ORF50 in cells

**Fig 9:**
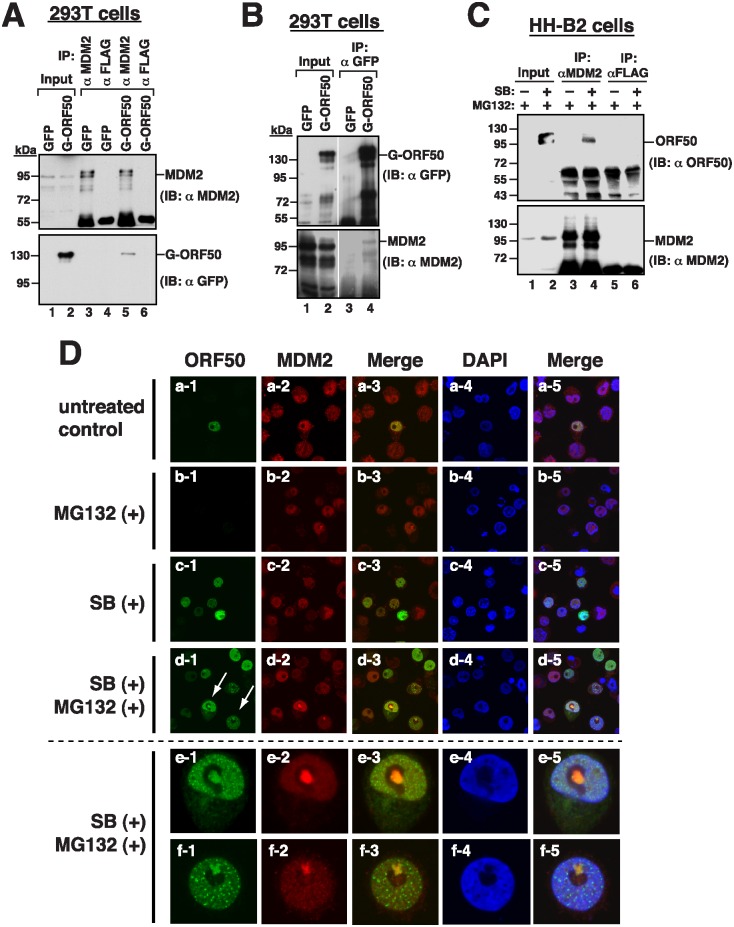
MDM2 interacts with ORF50 in cells. (A) 293T cells were transfected with the expression plasmid for GFP or GFP-ORF50. Cell lysates were harvested and immunoprecipitated with anti-MDM2 or anti-FLAG antibody. Cell lysates (input) and the resulting immunoprecipitates were probed with anti-MDM2 or anti-GFP antibody. (B) Same as in (A), except that the immunoprecipitation was carried out using anti-GFP antibody. (C) Coimmunoprecipitation of MDM2 and endogenous ORF50 in HH-B2 cells. HH-B2 cells were untreated or treated with 3 mM sodium butyrate (SB). At 6 hr after SB treatment, cells were then treated with MG132 (5 μM) for 20 hr. After the cell lysates were immunoprecipitated with anti-MDM2 or anti-FLAG antibody, the immunoprecipitated proteins were analyzed for the existence of MDM2 and ORF50 by immunoblotting. (D) Colocalization of MDM2 and ORF50 in HH-B2 cells. Confocal immunofluorescent analysis was performed using HH-B2 cells treated with MG132, SB or both SB and MG132. Unique ORF50/MDM2 colocalized complexes were observed in nucleolar areas when cells were treated with both SB and MG132 (d, arrows). The bottom sides (e and f) show high magnification of the selected cells from (d).

The involvement of MDM2 in the ORF50 protein stability prompted us to investigate whether MDM2 interacted with ORF50 *in vivo*. Accordingly, a lysate was prepared from 293T cells that had been transfected with a plasmid expressing GFP-ORF50. Immunoblotting revealed that anti-MDM2 antibody not only immunoprecipitated MDM2 but also coimmunoprecipitated GFP-ORF50 (Fig 9A, lane 5). However, neither was MDM2 nor GFP-ORF50 immunoprecipitated by anti-FLAG antibody (Fig 9A, lane 6). Meanwhile, anti-GFP antibody immunoprecipitated GFP-ORF50 and coimmunoprecipitated MDM2 (Fig 9B, lane 4). To further demonstrate the interaction in KSHV-infected cells, we treated HH-B2 cells with a combination of sodium butyrate (SB) and MG132 to activate the expression and stability of ORF50. We found that anti-MDM2 antibody immunoprecipitated MDM2 and coimmunoprecipitated ORF50 (Fig 9C, lane 4), demonstrating that MDM2 interacts with ORF50 in HH-B2 cells. Confocal microscopy analysis also demonstrated that ORF50 and MDM2 were colocalized in the nucleus in HH-B2 cells after lytic induction (Fig 9Dc–9Df). Particularly, the colocalized ORF50/MDM2 complexes could be also seen within nucleolar areas in HH-B2 cells after treatment with combinations of SB and MG132 (Fig 9Dd–9Df).

### Mapping the interaction domains between ORF50 and MDM2

**Fig 10:**
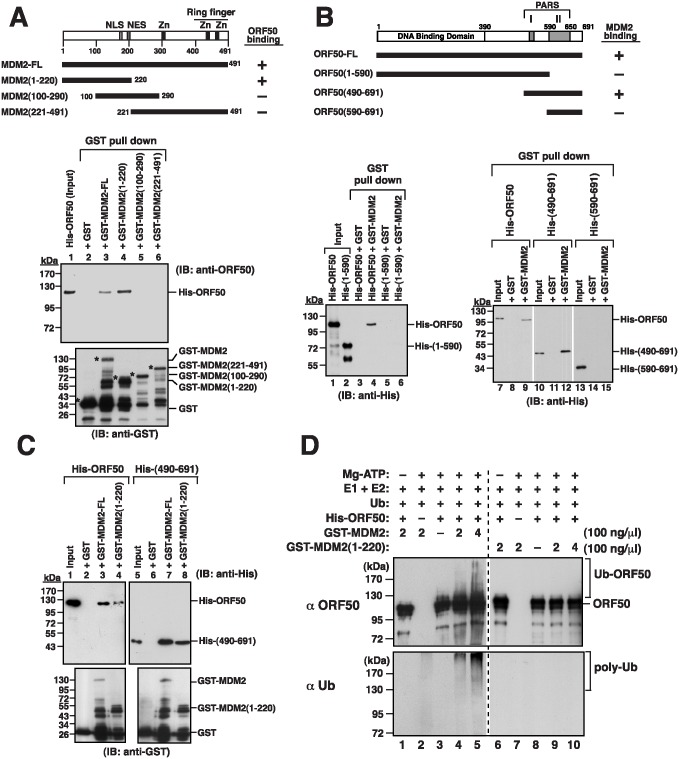
Mapping of the interaction domains between MDM2 and ORF50, and ubiquitination of ORF50 by MDM2 *in vitro*. (A) Defining the ORF50-interacting domain of MDM2. The purified His-tagged ORF50 (His-ORF50) was incubated with GST, GST-MDM2 or GST-MDM2 deletion mutants (1–220, 100–290 and 221–491) expressed in *E*. *coli*. Following pull-down with glutathione beads, the pull-down lysates were immunoblotted with anti-ORF50 or anti-GST antibody. (B) Defining the MDM2-interacting domain of ORF50. Glutathione beads conjugated to GST-MDM2 were incubated with His-ORF50 or His-ORF50 deletions as shown in the diagram. The GST pull-down precipitates were immunoblotted using anti-His antibody. (C) Detection of the interaction between GST-MDM2(1–220) and His-ORF50(490–691) in pull-down assay. (D) Effect of MDM2 on ORF50 ubiquitination *in vitro*. The *in vitro* ubiquitantion assay was performed using purified components as indicated. In the reactions, purified His-ORF50 protein was used at a final concentration of 120 nM and GST-MDM2 (or GST-MDM2(1–220)) at 100 nM or 200 nM. Reaction mixtures were analyzed by immunoblotting with anti-ORF50 or anti-Ub antibody.

This study further delineated the interaction regions in MDM2 and ORF50, using MDM2 fused to glutathione S-transferase (GST) and His-tagged ORF50 (His-ORF50). The integrity of both GST-MDM2 and His-ORF50 proteins was verified by immunoblotting analysis using at least two different antibodies that recognize either the N-terminal or C-terminal part of the target proteins (S7A and S7B Fig). Additionally, the purified His-ORF50 was demonstrated to be able to interact with its cellular partner RBP-Jκ expressed in 293T cells (S7C Fig). As shown in Fig 10A, immunoblot analysis revealed that His-ORF50 was pulled down by GST-MDM2-glutathione-Sepharose beads, but not pulled down by GST-glutathione-Sepharose beads, demonstrating that MDM2 interacts with ORF50 *in vitro*. We also expressed the MDM2 regions from amino acids 1 to 220, 100 to 290, and 221 to 491 that were fused to GST in *E*. *coli* (Fig 10A). Only MDM2(1–220), but not MDM2(100–290) and MDM2(221–491), pulled down His-ORF50 (Fig 10A, lanes 4–6). Meanwhile, different His-ORF50 deletions were also included to analyze their ability to interact with GST-MDM2 (Fig 10B). The mutant His-ORF50(1–590) could not be pulled down with GST-MDM2 (Fig 10B, lane 6), suggesting that the C-terminal portion of ORF50 is critical for the binding of MDM2. Further analysis revealed that His-ORF50(490–691), but not His-ORF50(590–691), was sufficient to interact with GST-MDM2 (Fig 10B, lanes 12 and 15). To further confirm the above results, pull-down experiments were performed to determine the interaction between His-ORF50(490–691) and GST-MDM2(1–220) (Fig 10C). Our results demonstrated that the PARS region (aa 490–691) of ORF50 directly interacts with the N-terminal domain (aa 1–220) of MDM2 (Fig 10C, lane 8).

### MDM2 and the ubiquitination of ORF50

To further evaluate whether MDM2 acts as an ubiquitin E3 ligase of ORF50, we performed an *in vitro* ubiquitination assay using purified components. The reconstitution reactions contained purified His-ORF50, GST-MDM2, E1, E2 (UbcH5B), Mg-ATP and ubiquitin. We did not detect ubiquitination of His-ORF50 by GST-MDM2 when Mg-ATP was omitted in the reaction (Fig 10D, upper panel, lane 1). Although ORF50 was previously identified as an E3 ligase and could auto-ubiquitinate itself in *vitro*, very low levels of ubiquitinated His-ORF50 were detected in the absence of GST-MDM2 in our reactions (Fig 10D, upper panel, lane 3). Poly-ubiquitination of His-ORF50 could be elevated with increasing concentrations of GST-MDM2 (Fig 10D, upper panel, lanes 4 and 5). However, replacing GST-MDM2 with GST-MDM2(1–220) in the reactions failed to increase the levels of ubiquitinated His-ORF50 (Fig 10D, upper panel, lanes 9 and 10). When the immunoblotting was carried out using anti-Ub antibody, we consistently found that the reactions containing both GST-MDM2 and His-ORF50 produced higher levels of ubiquitin conjugates than those with either GST-MDM2 or His-ORF50 alone (Fig 10D, bottom panel, lanes 2–5). These results supported that MDM2 serves as an ubiquitin E3 ligase of ORF50.

### Mutations in lysine residues and the stability of ORF50

**Fig 11:**
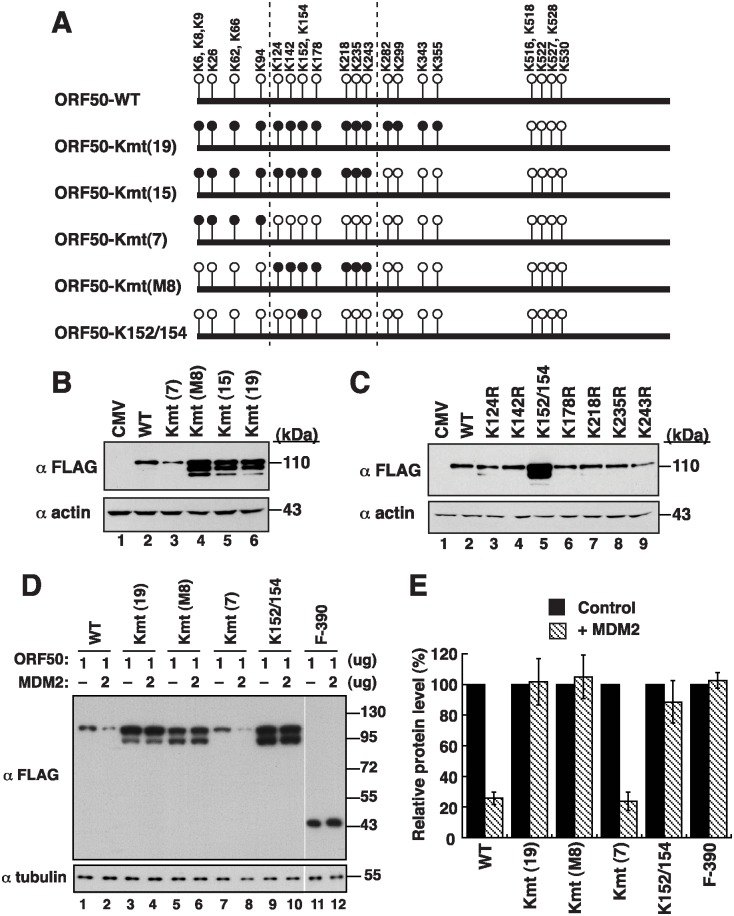
Lysine residues at positions 152 and 154 in ORF50 are critical for MDM2-mediated degradation. (A) Schematic diagram of ORF50 and ORF50 lysine mutants. The positions of 25 lysines in ORF50 are shown in the diagram (circles). Black circles represent the substitutions of lysine (K) with arginine (R) in F-ORF50. (B) Immunoblot analysis of lysine substitution mutants of ORF50. Cell lysates of 293T cells that were transfected with the indicated expression plasmids were probed with anti-FLAG antibody. (C) Effects of single- or double-lysine mutations from K124 to K243 (the middle 8 lysine clusters) on ORF50 abundance. (D) Susceptibility of ORF50 mutants to MDM2-mediated degradation. The expression plasmids encoding ORF50 mutants were cotransfected with an MDM2 expressing plasmid or control vector into 293T cells. The expression of these ORF50 mutants in the presence or absence of exogenous MDM2 was determined by immunoblotting with anti-FLAG antibody. (E) Relative protein levels of ORF50 mutants in the presence or absence of overexpressed MDM2. The bar graph summarizes densitometry data from three independent experiments. Error bars: standard deviation.

ORF50 contains 25 lysine residues that are clustered in two regions: 19 lysines in the N-terminal 356-aa region and 6 lysines in the PARS-I motif (Fig 11A). As shown above, the N-terminal 356-aa region is required for ORF50 polyubiquitination (Fig 7). To identify the lysine residues that are required for the control of ORF50 stability, we substituted the N-terminal 7 lysine (Kmt(7)), 15 lysines (Kmt(15)), 19 lysines (Kmt(19)), and the middle 8 lysines (Kmt(M8)) into arginine residues (Fig 11A). We found that F-ORF50-Kmt(15), F-ORF50-Kmt(19), and F-ORF50-Kmt(M8) were present in the cells more abundantly than F-ORF50 after transfection of 293T cells with the plasmids expressing these proteins (Fig 11B, lanes 4–6). However, F-ORF50-Kmt(7) was present in an amount that was comparable to that of F-ORF50 (Fig 11B, lanes 2 and 3). These results implicated the importance of lysine residues from K124 to K243 (the middle 8 lysine clusters) to the stability of ORF50. We also generated single or double mutations at the lysine residues in this region or in other regions (Fig 11C and S8 Fig). We found that mutating both K152 and K154 (Fig 11A, ORF50-K152/154) increased the stability of F-ORF50; mutating the other lysine residues did not affect the stability (Fig 11C and S8 Fig). More importantly, when compared to F-ORF50 or F-ORF50-Kmt(7), we found that mutants including F-ORF50-Kmt(19), F-ORF50-Kmt(M8) and F-ORF50-K152/154 were more resistant to MDM2-directed degradation (Fig 11D and 11E). These results showed that residues K152 and K154 are critically involved in MDM2-mediated degradation.

### Expression of MDM2 and ORF50 during KSHV reactivation

**Fig 12:**
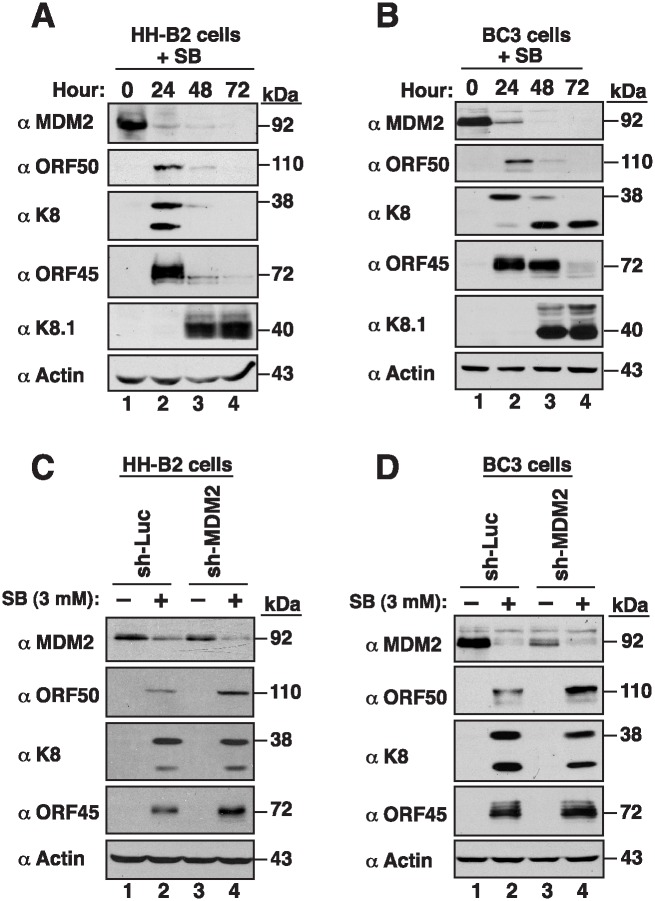
MDM2 negatively regulates ORF50 expression in KSHV-infected cells. The expression kinetics of MDM2 and viral lytic proteins (including ORF50, K8, ORF45, K8.1) were analyzed in HH-B2 (A) and BC3 cells (B) after treatment with sodium butyrate (SB). (C and D) The lentiviral vector-mediated MDM2 knockdown was performed in HH-B2 and BC3 cells. At 24 hr after lentiviral infection, HH-B2 or BC3 cells were either untreated or treated with SB for another 18 hr. The expression of MDM2, ORF50, K8 and ORF45 was determined by immunoblotting with the indicated antibodies.

To investigate how MDM2 affected ORF50 expression in KSHV-infected cells, the kinetics of MDM2 expression were examined in HH-B2 and BC3 cells after lytic induction. Upon treatment with SB, the early lytic proteins including ORF50, K8 and ORF45 were induced in HH-B2 and BC3 cells, followed by the expression of the late gene K8.1 (Fig 12A and 12B). By contrast, we found that the amount of MDM2 was reduced after lytic induction (Fig 12A and 12B). To further characterize the association between MDM2 and ORF50 expression, we performed MDM2 knockdown experiments and examined ORF50 expression at the early stage of lytic replication. Knockdown of MDM2 in HH-B2 or BC3 cells was not sufficient to induce ORF50 expression as detected by immunoblot analysis (Fig 12C and 12D, lanes 1 and 3), or by flow cytometry and quantitative RT-PCR (S9 Fig). The expressions of ORF50 and other viral lytic proteins were induced only in the cells treated with SB (Fig 12C and 12D, lanes 2 and 4). When compared to non-silencing controls, we found that MDM2 knockdown further enhanced the expression of ORF50 in HH-B2 or BC3 cells after SB treatment (Fig 12C and 12D, compare lanes 2 and 4). The enhanced ORF50 expression in MDM2-knockdown cells was not due to an increase in mRNA levels as detected by quantitative RT-PCR (S9C and S9D Fig), or a true increase in the numbers of ORF50-positive cells as detected by flow cytometry (S9A and S9B Fig). These results strongly suggested that MDM2 regulates ORF50 expression through a post-transcriptional mechanism, but not a transcriptional mechanism. Interestingly, although the expressions of ORF50 and its downstream target ORF45 were enhanced in MDM2-knockdown cells in response to SB, we did not observe a similar enhancement for another early lytic protein K8, implying that MDM2 may have profound effects on viral reactivation from latency.

## Discussion

The ORF50 protein is the key controller of KSHV reactivation in infected cells [8]. In the past, we have provided evidence that the abundance of ORF50 is controlled through a PARS region (PARS-I and PARS-II) at its C-terminal domain [10, 17]. Here, we extend our previous study by dissecting the molecular mechanism by which the abundance of ORF50 is modulated in cells. We demonstrate that the ubiquitin-proteasome degradation pathway is involved in the regulation of ORF50 abundance. Moreover, we identify that MDM2 E3 ligase binds to the PARS region in ORF50 and triggers the degradation of ORF50. All these findings suggest that MDM2 is a negative regulator of ORF50 expression and highlight a novel role of MDM2 in the maintenance of KSHV latency.

### ORF50 degradation is controlled through two critical steps

The PARS region that controls ORF50 abundance can be functionally divided into two components: PARS-I and PARS-II [10, 17]. Since ORF50 is a nuclear protein and the PARS-I motif overlaps with the NLS, we study the association between its subcellular localization and protein abundance. Our results show that all PARS-I mutants defective in nuclear entry are abundantly expressed in cells (Figs 2 and 4). The function of the PARS-I motif in the control of ORF50 abundance strongly correlates with its activity as a NLS. On the other hand, the PARS-II motif is not required for the nuclear localization of ORF50 (Fig 4). On the basis of analysis of different PARS-I and PARS-II mutants, we propose that the control of ORF50 degradation can be divided into two successive steps: i) nuclear translocation of the protein and ii) subsequent proteolysis in the nucleus. The translocation of ORF50 from the cytoplasm to the nucleus is mediated through the PARS-I (NLS) motif, whereas destabilization of ORF50 in the nucleus requires another PARS motif, PARS-II.

In agreement with previous reports [12, 14], our mutational analysis revealed that the functional NLS of ORF50 is the KKRK motif (aa 527–530), but not the KRK (aa 516–518) and RSK (aa 520–522) motifs, within the PARS-I motif (Fig 2). Based on the results obtained from the coexpression experiments (Figs 3 and 5), there are three important conclusions for the NLS-dependent nuclear transport of ORF50. Firstly, ORF50 constructs containing NLS (or PARS-I) appear to be dominant over the NLS mutant (e.g., F-ORF50(KK/EE)). Secondly, ORF50 has a tendency to oligomerize in the cytoplasm before the protein enters the nucleus. Thirdly, we confirmed that the multimerization domain of ORF50 is localized within the N-terminal region of ORF50 (Fig 5B). Besides the increase in the protein level, we noticed that retention of ORF50 mutants in the cytoplasm also substantially influences their phosphorylation status. Specially, the hypophosphorylated ORF50 (form B) tends to be more abundant than the hyperphosphorylated ORF50 (form A) in mutants including ORF50(KK/EE), ORF50(KK/AA) and ORF50(Δ514–530). The inefficient phosphorylation of ORF50-B in these mutants could be due to the saturation of available kinases or lack of specific kinases in the cytoplasm. Currently, the authentic relationship between ORF50 phosphorylation and protein stability is still unclear and requires further investigation. After the nuclear import of ORF50, we demonstrate that the PARS-II motif is required for ubiquitination and degradation of ORF50 protein (Figs 6 and 7). Although the C-terminal PARS-II-containing region from aa 590 to 691 is critically involved in ORF50 ubiquitination (Fig 7C), this region does not contain any lysine residues through which ubiquitin can be attached. Thus, we propose that the PARS-II motif may serve as the binding region for ubiquitin enzymes to control the degradation of ORF50 in the nucleus (Fig 7E).

### MDM2 interacts with ORF50 and promotes the degradation of ORF50

Human MDM2 is an E3 ubiquitin ligase, which is known as a negative regulator of p53 [18]. Herein, we show that ectopic overexpression of MDM2 specifically targets the degradation of full-length ORF50, but not PARS deletion mutants (Fig 8). As MDM2 interacts with ORF50 *in vitro* and *in vivo*, and promotes ORF50 ubiquitination *in vitro* (Figs 9 and 10), we strongly suggest that MDM2 is an E3 ubiquitin ligase for ORF50 in cells. Especially, during the course of treatment with SB and MG132 in HH-B2 cells, we found that ORF50 and MDM2 signals seem to colocalize in a structure associated with the nucleolus (Fig 9D). This event is very reminiscent of p53 regulation by MDM2 occurring in nucleoli [23]. It is thus important for future research to investigate whether nucleolar translocation is required for MDM2 to promote ORF50 degradation.

After mapping of the MDM2-binding region in ORF50, we found that the C-terminal region from aa 490 to 691 (PARS-I plus PARS-II), but not the PARS-II region only (from aa 590 to 691), is sufficient to interact with MDM2 (Fig 10B and 10C). These results indicate that the flanking region of PARS-II, which may be extended to the PARS-I, is also required for the binding of MDM2. Currently, we are not sure if the flanking region of PARS-II directly contacts MDM2 or if the flanking region is only critical for stabilizing the domain structure. Human MDM2 consists of 491 amino acids, which contains several well-characterized domains including the N-terminal p53-binding domain (aa 18–101) [18, 24]. Mapping the ORF50-interacting domain revealed that the N-terminal p53-binding domain of MDM2 is necessary for the interaction with ORF50 (Fig 10A). In addition to p53, the N-terminal region of MDM2 also interacts with p73, E2F1 and p63/p51, which share a conserved MDM2-binding motif [25]. After motif searching, we found that a similar MDM2-binding motif is present within the PARS-II region from aa 592 to 610 of ORF50 (S10 Fig). The importance of this predicted motif in ORF50 for MDM2 binding needs to be further verified. Besides the PARS regions, we demonstrate that the N-terminal 356-aa region is also critical for ubiquitination and degradation of ORF50 (Fig 7, F-ORF50(357–691)). Mutational analysis revealed that substitutions of the N-terminal 19 lysines, the N-terminal 15 lysines or the middle 8 lysines with arginines substantially increased their protein abundance (Fig 11A and 11B), and the resulting mutants were resistant to the degradation by MDM2 (Fig 11D and 11E). Particularly, residues K152 and K154 in the middle 8-lysine cluster of ORF50 were found to be the key sites for MDM2-mediated degradation (Fig 11D and 11E). These findings strongly suggest that lysine residues at positions 152 and 154 in the N-terminal region of ORF50 are potential ubiquitin acceptor sites for ORF50 degradation (Fig 7E).

In the *in vitro* ubiquitination assay, we showed that purified GST-MDM2 could promote ubiquitination of His-ORF50 protein expressed in *E*. *coli* (Fig 10D). However, although ORF50 protein reportedly possesses an E3 ubiquitin ligase activity and can auto-ubiquitinate itself in *vitro* [9, 16, 26], very little E3 ligase function for the purified His-ORF50 was detected in our experimental conditions (Fig 10D, lane 3). This discrepancy may be due to the use of different ORF50 constructs (GST-ORF50 vs. His-ORF50) [9] or ORF50 protein purified from different sources (insect cells vs. bacteria) [16, 26]. Additionally, instead of UbcH5A, we used UbcH5B as an E2 ubiquitn-conjugating enzyme in our *in vitro* ubiquitination reactions. Although UbcH5A and UbcH5B have 89% amino acid sequence identity, the reason we used UbcH5B in our *in vitro* ubiquitination assay is that UbcH5B is the main physiological E2 for MDM2 [27]. Further studies may be required to clarify the precise role of different E2s in supporting the ubiquitin ligase activity of ORF50 protein.

### Roles of MDM2 during latency and reactivation of KSHV

Previously, Ye et al. [20] have demonstrated that Nutlin-3, a small-molecule MDM2 inhibitor, induces the expression of viral lytic genes in their KS mouse model. Further studies by Balistreri et al. [21] also revealed that depletion of MDM2 promotes KSHV lytic replication. In our study, we show that MDM2 functions to target ORF50 degradation. All of these findings point out that MDM2 is a negative regulator of KSHV reactivation from latency. In other words, MDM2 may have an important role to establish or maintain viral latency in KSHV infected cells. Consistent with this notion, Petre et al. [28] have previously shown that MDM2 mRNA levels are highly expressed in PEL tumor samples as compared to that in other B-cell tumors including Burkitt’s lymphoma and different types of diffuse large B-cell lymphoma, after the examination of transcriptional profiles from over 60 B-cell tumors.

During the latency or during a primary infection, viral lytic gene transcripts including ORF50 mRNA could be detected at low levels in infected cells [29]. Theoretically, low-level expression of ORF50 in these cells may auto-stimulate its own promoter through its association with other transcriptional regulators including Oct-1, C/EBPα, and RBP-Jκ [30–32]. Once a threshold level of ORF50 expression is achieved, the lytic cascade will proceed to completion. In addition to the transcriptional repression of the *orf50* gene, rapid degradation of ORF50 protein is also an important strategy to establish or maintain viral latency. MDM2 may function to lower basal level of ORF50 protein in these infected cells, thus keeping viral latency. Previously, Chen et al. [33] and Santag et al. [34] have showed that LANA, the key controller of KSHV latency, interacts with the MDM2/p53 complex in cells. They demonstrate that formation of the LANA-MDM2-p53 complex is relevant to maintain viral latency [34]. Whether the LANA-MDM2-p53 complex is preferentially involved in ORF50 degradation still needs to be further investigated.

Upon viral reactivation induced by sodium butyrate, we found that the levels of MDM2 declined gradually in HH-B2 and BC3 cells (Fig 12A and 12B). Although the downregulation of MDM2 is not sufficient to initiate lytic reactivation, the decreased MDM2 expression may favor the progression of lytic replication (Fig 12C and 12D). During the onset of viral reactivation, we think that the balance between ORF50 and MDM2 expression may be critical to determining the switch between latency and the lytic cycle. Despite decreased expression of MDM2 at the early lytic stages, the residual MDM2 may still retain its negative function on ORF50 expression. Jaber et al. [35] previously reported that vSP1 (viral small peptide 1), an immediate-early gene product encoded by KSHV T3.0 transcript, could increase ORF50 stability by binding to the PARS-II region of ORF50 in cells [35]. As both vSP1 and MDM2 bind to the same PARS region of ORF50, the expression of vSP1 during lytic reactivation may facilitate the dissociation of ORF50 from MDM2. Intriguingly, during the late stage of lytic replication, we consistently observed that levels of ORF50 protein started to decline from 24 to 48 hr after HH-B2 or BC3 cells were treated with sodium butyrate (Fig 12A and 12B). At the late lytic phase, the downregulation of ORF50 may be unrelated to MDM2 because the level of MDM2 is low under the conditions. It is possible that other viral lytic proteins or their elicited cellular factors manipulate the downregulation of ORF50 in PEL cells during the late phase of lytic replication, probably via a transcriptional or/and post-transcriptional mechanism.

In summary, we reveal the molecular mechanism of ORF50 degradation and demonstrate that MDM2 is involved in the degradation of ORF50. Since ORF50 is the key controller of KSHV reactivation, understanding the turnover of ORF50 may provide new insights into the switch between latency and lytic reactivation of KSHV.

## Materials and Methods

### Cell cultures and transfections

293T, HEK293 and HKB5/B5 [10, 11] cells were cultured in Dulbecco's modified Eagle's medium (DMEM) supplemented with 10% fetal calf serum (FBS). HH-B2 [7] and BC3 [36] are PEL cell lines infected with KSHV. Both HH-B2 and BC3 cells were cultured in RPMI 1640 medium supplemented with 15% FBS. Transient transfection experiments were performed using Lipofectamine 2000 according to the manufacturer’s instructions.

### Plasmid construction

Several plasmids that express wild-type FLAG-tagged ORF50 (pCMV-FLAG-ORF50) and its mutants used in the study have been described previously [17]. For generating ORF50 deletion mutants, specific DNA fragments of the *orf50* gene were amplified from pCMV-FLAG-ORF50 by PCR and then cloned into pFLAG-CMV-2 (Sigma-Aldrich). The QuickChange site-directed mutagenesis kit (Stratagene) was used to introduce point mutations at the indicated sites of the *orf50* gene. Plasmids encoding GFP-ORF50 and its mutant derivatives were constructed by inserting appropriate *orf50* gene fragments into pEGFP-C2 (Clontech). To construct the plasmids expressing GFP-ORF50+NLS and GFP-ORF50(KK/EE)+NLS, double-stranded oligonucleotides corresponding to the coding sequence of the SV40 NLS were first inserted into pEGFP-C2, and then specific DNA fragments of the *orf50* gene were cloned upstream of the SV40 NLS coding sequence. The coding sequence of the SV40 NLS is 5’-ATGGGCCCTAAAAAGAAGCGTAAAGTC, which encodes a peptide MGPKKKRKV. The plasmid pCMV6-XL-MDM2, an MDM2 expression vector, was purchased from OriGene (Rockville, MD). Plasmids that expressed GST-MDM2, GST-MDM2(1–220), GST-MDM2(100–291), and GST-MDM2(221–491) were generated by inserting corresponding MDM2 DNA fragments into pGEXT-4T1 (Amersham Biosciences, NJ). For expression of the recombinant His-tagged ORF50 or its deletion mutants in bacteria, DNA fragments encoding specific ORF50 domains were inserted into either pET-32a or pET-22b (Novagen).

### Confocal immunofluorescence analysis

293T cells (1 x 10^5^) were grown on coverslips in 6-well tissue culture plates and transiently transfected with plasmids for 24 hr. After transfection, cells were fixed with 4% paraformaldehyde in phosphate-buffered saline (PBS) at 4°C for 30 min, and then permeabilized with 0.1% Triton X-100 in PBS for 7 min. To detect the FLAG-tagged proteins, cells were initially incubated with blocking solution (1% BSA) at room temperature for 1 hr, and then treated with polyclonal rabbit anti-FLAG antibody (F7425; Sigma-Aldrich) for 2 hr. After three washing steps, Alexa Fluor 594-conjugated goat anti-rabbit IgG polyclonal antibody (A11037; Molecular Probes) was added to treat the cell samples. Staining with 4′-6-diamidino-2-phenylindole (DAPI) was performed at room temperature for 15 min. Cells were mounted in Citifluor (Agar Scientific) and observed under a confocal laser-scanning microscope (model LSM 510 META; Zeiss) [37]. To determine the localization of endogenous ORF50 and MDM2, HH-B2 cells were first treated with 3 mM sodium butyrate (SB). At 6 hr after SB treatment, MG132 was added at a final concentration of 5 μM and the cells were incubated for another 20 hr. Cells were then fixed and stained using antibodies specific to MDM2 (M4308; Sigma-Aldrich) and ORF50 [38].

### Immonoblot analysis

Cell extracts were prepared and mixed with 3x sodium dodecyl sulfate (SDS) gel loading buffer. After the mixtures were boiled for 5 min, proteins were resolved on an 8% to 12% polyacrylamide gel. The proteins on the gels were then transferred onto a PVDF membrane (Bio-Rad) and were probed with specific antibodies. The anti-ORF50 polyclonal antibody used in the study was generated in our laboratory using purified ORF50(333–691) as an immunogen. Antibodies to FLAG (F3165; Sigma-Aldrich), tubulin (T5168; Sigma-Aldrich), GFP (ab290; Abcam), HA (H9658; Sigma-Aldrich), MDM2 (M4308; Sigma-Aldrich), K8 (sc-57889; Santa Cruz), ORF45 (sc-53883; Santa Cruz) and actin (sc-47778; Santa Cruz) were purchased commercially.

### Ubiquitination of ORF50

293T cells (1×10^7^) were transfected with pCMV-HA-Ub and pCMV-FLAG-ORF50 or ORF50 mutant constructs. At 16 hr posttransfection, cells were further treated with 5 μM MG132 for 24 h to stabilize the ubiquitinated proteins. Cells were harvested and lysed in 200 μl of the ubiquitination protective buffer (a solution obtained by mixing buffer-I and buffer-II in a ratio of 1:3; Buffer-I contains 5% SDS, 150 mM Tris-HCl [pH 6.7] and 30% glycerol; Buffer-II contains 25 mM Tris-HCl [pH 8.2], 50 mM NaCl, 0.5% NP-40, 0.1% sodium azide and 0.1% SDS) [39, 40]. After sonication and incubation at 95°C for 20 min, supernatants were collected by centrifugation and then diluted with 1800 μl of PBS containing 0.5% Nonidet P-40. Immunoprecipitation was performed by mixing protein lysates with anti-HA (H9658; Sigma-Aldrich) or anti-FLAG antibody (A8592; Sigma-Aldrich) for 6 hr at 4°C. The protein mixtures were then incubated with protein A/G agarose beads (Upstate) for another 1.5 h at 4°C. Bound proteins were extracted and were analyzed by immunoblotting.

### Protein stability assay

293T cells were cotransfected with pCMV-FLAG-ORF50 and pCMV6-XL-MDM2 or vector control. At 19 hr posttransfection, cells were treated with 75 μg/ml cycloheximide (Sigma-Aldrich) to inhibit de novo protein synthesis. At indicated time points after treatment with cycloheximdie, cells were harvested and subjected to immunoblot analysis. Relative levels of F-ORF50 normalized to α-tubulin were quantified by densitometry.

### Coimmunoprecipitation

293T cells were transfected with pEGFP-C2 or pCMV-GFP-ORF50 for 30 hr, whereas HH-B2 cells were untreated or treated with 3 mM SB for 6 hr, and then incubated with 5 μM MG132 for another 20 hr. Cell samples were harvested and resuspended in radioimmunoprecipitation assay (RIPA) buffer (50 mM Tris-HCl [pH 7.6], 0.1 M NaCl, 5 mM EDTA, 0.5% Triton X-100, 0.5% NP-40, 0.1% sodium deoxycholate, and 1mM phenylmethylsulfonyl fluoride) [41]. Proteins in the lysate were immunoprecipitated using anti-MDM2, anti-GFP or anti-FLAG antibody. After extensive washing, the immunoprecipitated proteins were analyzed by immunoblotting with specific antibodies.

### GST pull-down assay

GST-MDM2 fusion proteins and His-tagged ORF50 proteins were expressed in *E*. *coli* BL21(DE3). The bacterial cells expressing recombinant proteins were harvested and were lysed in NETN buffer (20 mM Tris-HCl (pH 8.0), 100 mM NaCl, 1 mM EDTA, 0.5% NP-40) [39]. In the pull-down assay, total bacterial lysates (5 mg) containing GST or GST-MDM2 (or GST-MDM2 deletions) were initially incubated with 30 μl glutathione-Sepharose 4B for 2 h at 4°C. After washing, purified GST or GST fusion protein was mixed with protein lysates containing His-ORF50 or His-ORF50 deletions for another 1 h at 4°C. Proteins that were pulled down by glutathione beads were extracted and subjected to immunoblot analysis.

### *In vitro* ubiquitinylation assay

The complete reaction mixture (25 μl) contained 100 nM E1 (Enzo, BML-UW9410), 2.5 μM E2 (UbcH5B) (Enzo, BML-UW9060), 11 μM Ub (sigma, SI-U6253), 5 mM Mg-ATP (Enzo, BML-EW9805), His-ORF50 (120 nM) and GST-MDM2 (100 or 200 nM) in the ubiquitinylation buffer (Enzo, BML-KW9885). In some reactions individual components were omitted or substituted as indicated. The *in vitro* ubiquitinylation reaction was incubated at 37°C for 90 minutes.

### Lentivirus-based knockdown

The preparation of shRNA lentiviral particles has been described previously [42]. All RNAi reagents were obtained from the National RNAi Core Facility Platform at the Institute of Molecular Biology/Genomic Research Center, Academia Sinica. The target sequence of the *MDM2* shRNA is 5’-ATTATCTGGTGAACGACAAAG. For lentiviral transduction, HH-B2 or BC3 cells were infected with the collected lentiviruses in the presence of polybrene at a final concentration of 8 μg/ml. After 24 hr post-infection, HH-B2 and BC3 cells were left untreated or treated with 3 mM SB for another 18 hr.

## Supporting Information

S1 Fig ORF50 is a hyperphosphorylated protein in cells. Protein extracts of HKB5/B5 cells transfected with pCMV-FLAG-ORF50 or pCMV-FLAG-ORF50(KK/EE) were treated with calf intestinal phosphatase (CIP) or lambda protein phosphatase (λPPase). After 1.5 hr incubation at the indicated temperature, the treated cell extracts were analyzed by immunoblotting using anti-FLAG antibody. ORF50-A (110 kDa): a hyperphosphorylated form of ORF50; ORF50-B (90 kDa): a hypophosphorylated form of ORF50.
(EPS)

S2 Fig Confocal fluorescent images of ORF50 and its PARS-I mutants in 293T cells. 293T cells were transiently transfected with the indicated plasmids for 24 hr. Cells were fixed, permeabilized, and probed with anti-FLAG antibody. Images were taken with the same settings on the confocal microscopy. The observed degree of ORF50 abundance in 293T cells is evaluated by “+”. N: nucleus; C: cytoplasm; N/C: both nucleus and cytoplasm.
(EPS)

S3 Fig Confocal fluorescent images of ORF50 and its C-terminal deletion mutants in 293T cells. After transfection with the indicated plasmids for 24 hr, cells were harvested, fixed, permeabilized, and probed with anti-FLAG antibody. All images were taken with the same settings on the confocal microscopy. The observed degree of the abundance of ORF50 mutants in 293T cells is evaluated by “+”. N: nucleus; C: cytoplasm; N/C: both nucleus and cytoplasm.
(EPS)

S4 Fig Multiple HRP-conjugated anti-immunoglobulin antibodies cross-react with ORF50 protein in immunoblotting experiments. Total protein lysates of 293T cells that were transfected with plasmids expressing wild-type F-ORF50 or its deletion mutants were prepared for immunoblotting analysis. Each lane in the assay contains 20 μg of lysate protein. An HRP-conjugated anti-FLAG antibody (A) and various HRP-conjugated anti-immunoglobulin antibodies (B-G) were used in immunoblotting analysis. The epitope that is recognized by these secondary antibodies is mapped to the C-terminal region from aa 650 to 691 of ORF50 protein.
(EPS)

S5 Fig Screening for candidate E3 ubiquitin ligases that modulate ORF50 stability. (A and B) Effect of dominant-negative cullin expression on ORF50 stability. 293T cells were cotransfected with pCMV-FLAG-ORF50 and the indicated plasmids expressing dominant negative cullins (pcDNA3-DN-hCUL1-FLAG, pcDNA3-DN-hCUL2-FLAG, pcDNA3-DN-hCUL3-FLAG, pcDNA3-DN-hCUL4A-FLAG, pcDNA3-DN-hCUL4B-FLAG, and pcDNA3-DN-hCUL5-FLAG). All plasmids encoding dominant negaive cullins were gifts from Dr. Wader Haper’s lab (Addgene plasmid #15818, #15819, #15820, #15821, #15822 and #15823). The expression levels of F-ORF50 and dominant negative cullins in cells were determined by immunoblotting using anti-FLAG antibody. (C and D) Effect of UBE3A or GFP-RNF4 expression on ORF50 abundance. Increasing amounts of an UBE3A or GFP-RNF4 expression plasmid were cotransfected with the F-ORF50 expression plasmid in 293T cells. At 24 hr after cotransfection, immunoblotting analysis was carried out to determine the expression of F-ORF50, UBE3A and GFP-RNF4.
(EPS)

S6 Fig MDM2 knockdown in 293T cells increases expression of wild-type ORF50, but not the C-terminal truncated mutant. At 24 hr after transduction with lentiviruses expressing sh-Luc or sh-MDM2, cells were then transfected with the plasmid expressing either F-ORF50 or F-590 for another 24 hr. The expression of ORF50 proteins and MDM2 was determined by immunoblotting.
(EPS)

S7 Fig Verification of the integrity of purified GST-MDM2 and His-ORF50 expressed in *E*. *coli*. (A) Immunoblotting analysis of GST-MDM2 using different antibodies (M4308, sigma; 19058-1-AP, Proteintech) that recognize either the N-terminal or C-terminal part of MDM2. (B) Immunoblotting analysis of His-ORF50 using different polyclonal rabbit antibodies that recognize the N-terminal ORF50(1–590) or C-terminal ORF50(333–691) region. Both polyclonal anti-ORF50 antibodies were generated in our laboratory. An unrelated His-tagged protein (FenB protein of *B*. *subtilis*) was used a negative control. (C) Detection of the interaction between His-ORF50 and cellular RBP-Jκ in pull-down assay. Total protein lysates of 293T cells that were transfected with the plasmid expressing FLAG-tagged RBP-Jκ (F-RBP-Jκ) were used in pull-down assay. Cellular F-RBP-Jκ was pulled down by His-ORF50-Ni-NTA resins, but not pulled down by control Ni-NTA resins.
(EPS)

S8 Fig Protein abundance of ORF50 lysine mutants. (A) Diagram of the positions of all 25 lysine residues in ORF50. The N-terminal 356-aa region contains 19 lysine residues and is divided into three clusters, including N7, M8 and C4. (B-D) Immunoblotting analysis of K-to-R mutants in 293T cells. Cells were transfected with the indicated plasmids for 24 hr. Cell lysates were then immunoblotted using anti-FLAG antibody. Note that only the mutant with substitutions at residues K152 and K154 (K152/154) shows an elevated protein expression.
(EPS)

S9 Fig Effect of MDM2 knockdown on ORF50 expression in HH-B2 and BC3 cells. (A and B) Flow cytometric analysis of ORF50 and MDM2 expression in HH-B2 and BC3 cells. The lentiviral vector-mediated MDM2 knockdown was performed in HH-B2 and BC3 cells. At 24 hr after lentiviral infection, HH-B2 or BC3 cells were either untreated or treated with SB for another 18 hr. Cells were subjected to flow-cytometry analysis using anti-MDM2 antibody (M4308; Sigma-Aldrich) and anti-ORF50 antibody. (C and D) Quantitative RT-PCR analysis of ORF50 and MDM2 mRNA levels in HH-B2 and BC3 cells. Total RNAs were isolated from the above treated cells and then were subjected to quantitative RT-PCR analysis as described previously [38]. The relative levels of ORF50 and MDM2 mRNAs in the treated cells were calculated by normalizing with the levels of 18S rRNA.
(EPS)

S10 Fig Sequence alignment of MDM2-binding motifs and the predicted motif in the PARS-II region of ORF50. The MDM2-binding motifs found in p53, p73, E2F1 and p63/51 are included in the alignment. Black dots on top indicate the residues in p53 that make contact with MDM2.
(EPS)
